# Propensity-matched analysis of the efficacy of olanexidine gluconate versus chlorhexidine-alcohol as an antiseptic agent in thoracic esophagectomy

**DOI:** 10.1186/s12893-022-01480-8

**Published:** 2022-01-22

**Authors:** Takeo Fujita, Naoya Okada, Takuji Sato, Kazuma Sato, Hisashi Fujiwara, Takashi Kojima, Hiroyuki Daiko

**Affiliations:** 1 Division of Esophageal Surgery, National Cancer Center Hospital East, Kashiwa, Japan; 2 Division of Gastrointestinal Oncology, National Cancer Center Hospital East, Kashiwa, Japan; 3 Division of Esophageal Surgery, National Cancer Center Hospital, Tokyo, Japan

**Keywords:** Esophagectomy, Olanexidine gluconate, Chlorhexidine-alcohol, Propensity-matched analysis

## Abstract

**Background:**

In the present matched-cohort study, we investigated the efficacy of olanexidine gluconate in comparison with chlorhexidine-alcohol as an antiseptic agent in thoracic esophagectomy.

**Methods:**

A total of 372 patients with esophageal cancer who were scheduled to undergo thoracic esophagectomy between 2016 and 2018 were assigned to one of two groups based on the preoperative antiseptic agent used in thoracic esophagectomy. We investigated the incidence of surgical site infectious complications in the propensity-matched cohort.

**Results:**

Based on the propensity score, 116 patients prepared with 1.5% olanexidine gluconate and 114 patients prepared with 1.0% chlorhexidine-alcohol as surgical skin antisepsis were selected. No significant intergroup differences were observed with respect to incisional surgical site infection (0.8% in the olanexidine group versus 0.8% in the chlorhexidine group) and deep fascial/organ space surgical site infection (1.7%/10.3% in the olanexidine group versus 3.5%/15.7% in the chlorhexidine group, *p* = 0.39/*p* = 0.03). Notably, the respective incidences of surgical site infection except anastomotic leakage were 1.7% and 7.0% in the olanexidine and chlorhexidine groups (*p* = 0.04).

**Conclusions:**

Olanexidine gluconate was well tolerated and significantly reduced incidence of surgical site infection except anastomotic leakage in comparison with chlorhexidine-alcohol as an antiseptic agent in thoracic esophagectomy with three-field lymph node dissection.

## Background

The use of optimal skin antiseptic agents is of critical importance, especially during traumatic surgical procedures [1, 2]. One of the most common surgical complications is surgical site infection (SSI), particularly in gastrointestinal surgery. SSI prolongs the postoperative hospital stay, which increases the medical cost. Multiple management protocols are followed to prevent the SSI. Of these, appropriate prophylactic antibiotics and skin antiseptic agents are the most important to minimize the risk of SSI. The major preoperative antiseptic agents that have been widely used as skin disinfectants throughout the world for a long time are chlorhexidine and povidone iodine [3, 4]. Recent clinical studies suggest that chlorhexidine-alcohol reduces the incidence of postoperative SSI compared with povidone iodine [5, 6].

Olanexidine (Olanedine; Otsuka Pharmaceutical Factory, Inc., Tokushima, Japan), containing 1.5% olanexidine gluconate, is a new skin antiseptic agent that was approved in Japan in 2015. This newly developed antiseptic agent is a biguanide disinfectant with a similar structure to chlorhexidine, with a wide range of antimicrobial activity. Olanexidine is particularly effective against methicillin-resistant *Staphylococcus aureus*, vancomycin-resistant *Enterococcus*, *Pseudomonas aeruginosa*, and even *Serratia marcescens* compared with chlorhexidine [7, 8]. Recent randomized control studies have demonstrated that olanexidine significantly reduces SSI compared with povidone iodine in clean-contaminated surgery [5, 9]. However, it is unknown whether olanexidine is superior to chlorhexidine-alcohol in preventing SSI in clean-contaminated surgery. In the present study, we retrospectively investigated the incidence of SSI and performed a propensity matched-analysis to compare the efficacy of olanexidine gluconate versus chlorhexidine-alcohol in clean-contaminated surgery, particularly in patients with esophageal cancer undergoing thoracic esophagectomy.

## Methods

### Study design

This study was a retrospective, single-center, two-arm cohort study performed between 2016 and 2018 at the National Cancer Center Hospital East, Japan. Eligible patients were assigned to receive skin disinfection with either 1.5% olanexidine gluconate (Olanedine™; Otsuka Yakuhin Kogyo, Tokyo, Japan) or 1.0% chlorhexidine-alcohol (Sterichlone™; Kenei Seiyaku, Tokyo, Japan) 30 min before surgery, and 372 consecutive patients were analyzed. Written informed consent was obtained from all patients. The study was approved by the Committee for ethics of the National Cancer Center (Japan) (approval number #2018-332). Also, this study confirms to the provisions of the Declaration of Helsinki (as revised in Tokyo 2004).

### Patients

Patients who underwent thoracic esophagectomy for esophageal cancer at the National Cancer Center Hospital East, Japan, were investigated. Preoperative diagnoses were based on imaging studies, including upper gastrointestinal studies, endoscopy, and conventional cross-sectional imaging (computed tomography). Histological evaluation of endoscope-guided biopsy specimens was performed in all cases. Data on the preoperative stage of the disease, histopathological findings, surgical procedures performed, and outcomes were collected from the patient medical records, as described previously [10].

In all cases, thoracic esophagectomy was performed under the direction of the regular attending surgeon. For transthoracic esophagectomy, subtotal resection of the esophagus was performed with three-field regional lymph node dissection, regardless of the tumor stage. In the thoracoscopic approach, we preserved the azygos arch and the right bronchial artery. In contrast, the azygos arch was transected in all patients who underwent the thoracotomy approach. The laparoscopic approach was principally used, except in cases with bulky lymph node metastasis or previous history of abdominal surgery via laparotomy. The esophagus was usually reconstructed with a gastric tube via the retrosternal route; right hemi-colic reconstruction was performed via the posterior sternal route when gastrectomy of the remnant stomach was required, as described previously [10].

### Anesthesia and intraoperative management during thoracic esophagectomy

The standard institutional anesthetic practice for thoracic esophagectomy was modified to enable the development of intraoperative core hypothermia in the present study. In brief, upon patient arrival in the operating room, routine monitoring was applied, which included electrocardiography, noninvasive blood pressure monitoring, pulse oximetry, and capnography. Before the induction of anesthesia, an epidural catheter was inserted at the fifth to sixth thoracic interspace, and placed 5 cm beyond the introducing needle tip, as described previously [10].

Anesthesia was induced with 1.5–2.5 mg/kg propofol, 1–2 μg/kg fentanyl, and 0.1 mg/kg vecuronium. Anesthesia was maintained with 3% end-tidal sevoflurane in oxygen until tracheal intubation. Anesthesia was then maintained with 2% end-tidal sevoflurane at 40% oxygen (air/oxygen mixture at 4 L/min) supplemented with doses of fentanyl and vecuronium. A heat- and moisture-exchanging filter was positioned between the endotracheal tube and the breathing circuit, as described previously [10].

### Definition of surgical complications

SSI was judged in accordance with the definition established by the Surgical Wound Infection Task Force 1, and included infections at the incision site or organ/space manipulated during operative intervention. Remote infections were not included in the definition of an SSI, with the exception of bloodstream infections related to a SSI. Symptomatic remote infections were also included in the analysis. Among the remote infections, respiratory infection was defined as the presence of new or progressive infiltrates on chest radiographs, plus at least two of the following signs of respiratory tract infection: temperature > 38 °C, purulent sputum, leukocytosis of > 1 × 10^4^/mm^3^ or leukopenia of < 4 × 10^3^/mm^3^, and signs of inflammation on auscultation, as described previously [10].

### Perioperative management

Perioperative management was performed with the same clinical management pathway (CMP) for all patients, regardless of the type of abdominal approach, as described previously [10]. All patients received enteral nutrition through a nasal feeding tube until the start of oral intake on postoperative day (POD) 6. In brief, fluid balance was achieved through a peripheral line, with additional enteral feeding on POD 1. Enteral nutrition was discontinued after the absence of anastomotic leakage was confirmed on POD 6. Perioperative antimicrobials are used only the surgery Cefmetazole sodium.

Perioperative management was performed by the same clinical staff in the same environment using the ICU and subsequent ward-based facilities. The same principles of care were applied to both groups. The CMP was applied in both the ICU (POD 1 and 2) and the surgical ward (POD 3 and later). In brief, the endotracheal tube was removed from all patients in the operating room or immediately upon arrival in the ICU. Patients remained in the ICU for 1 day after surgery. On POD 6, a radiographic contrast agent swallow examination was performed to evaluate the anastomosis and any passage problems. If this examination showed no leakage or obstruction, the nasogastric tube was removed and oral intake was initiated in accordance with the postoperative diet program. In the absence of any complications, the patient was enrolled in the postoperative rehabilitation program and discharged on POD 12–20, as described previously [10].

If there were any abnormal clinical findings such as hypoxia, leukocytosis, or abnormal pleural drainage during the course of the postoperative CMP, computed tomography and radiographic examinations were performed to diagnose and optimally manage the abnormality as soon as possible as described previously [10].

### Statistical analysis

Propensity score matching was used to assemble two comparable groups. The covariates were preoperative treatment, thoracic surgical approach, abdominal surgical approach, clinical stage, ASA Grade, and type of antibiotics. Each patient in the olanexidine gluconate group was matched to a patient in the chlorhexidine-alcohol group who had the closest propensity score using simple 1:1 matching without replacement. To prevent poor matches, a caliper of 0.20 of the standard deviation of the logit of the propensity score was used. Intergroup differences were analyzed using the Chi-squared test and the Mann–Whitney U-test. A *p* value < 0.05 was considered to indicate a statistically significant difference. All statistical analysis were performed using R calculation software.

## Results

Of the 372 included patients, the antiseptic agent was chlorhexidine-alcohol in 178 patients, and 1.5% olanexidine gluconate in 194 patients. Olanexidine gluconate was well tolerated and no adverse events specific to its administration were observed. All patients were successfully treated using the CMP without any problems. There were no significant differences between the chlorhexidine-alcohol and olanexidine gluconate groups with respect to baseline patient characteristics, including age, sex, body mass index, ASA grade, preoperative treatment, and clinical stage of the disease before adjusting (Table 1). Operative procedures and variables during esophagectomy in all 372 patients are shown in Table 2. There were no significant differences between groups in the thoracic and abdominal surgical approaches, total operative duration, total amount of intraoperative blood loss before adjusting (Table 2). There were no significant intergroup differences regarding the immediate pre- and postoperative skin reaction before and after esophagectomy (data not shown). A summary of the short-term outcomes after esophagectomy in all 372 patients before adjusting are presented in Table 3. There were no significant differences between groups in the 30-day incidence of SSI (16.4% in the chlorhexidine-alcohol group versus 17.4% in the olanexidine gluconate group, *p* = 0.81), incidence of anastomotic leakage (12.3% in the chlorhexidine-alcohol group versus 12.3% in the olanexidine gluconate group, *p* = 0.99), or in-hospital deaths (*p* = 0.95). The success rates of the CMP were similar in both groups (72.1% in the chlorhexidine-alcohol group versus 78.0% in the olanexidine gluconate group; *p* = 0.18).

**Table 1:** Patients’ characteristics

Variables	Olanexidine group (n = 194)	Chlorhexidine group (n = 178)	p value
Age (mean ± S.D.)	68.5 ± 4.4	67.6 ± 5.9	0.21
Gender (M:F)	158:36	151:27	0.38
Body Mass Index (mean ± S.D.)	21.5 ± 2.6	21.7 ± 2.8	0.55
ASA Grade			0.39
Grade 1	78	64	
Grade 2	116	114	
Pre-operative treatment			0.43
Chemotherapy	95	92	
Chemo-radiation	17	12	
Clinical stage (UICC 7th)			0.65
Stage I	47	51	
Stage II	49	48	
Stage III	77	68	
Stage IV	17	11

**Table 2:** Operative procedure and variables during esophagectomy

Variables	Olanexidine group (n = 194)	Chlorhexidine group (n = 178)	p value
Type of surgical approach			0.43
Thoracoscopic	161	153	
Thoracotomy	33	25	
Type of surgical approach			0.37
Laparoscopic	165	157	
Laparotomy	29	21	
Total time of procedure (min)	383.6	368.1	0.22
Total amount of blood loss during surgery (ml)	183.2	211.5	0.44

**Table 3:** Postoperative events and success rate of the CMP

Variables	Olanexidine group (n = 194)	Chlorhexidine group (n = 178)	p value
30-days SSI (%)	32 (16.4)	31 (17.4)	0.81
Anastomotic leakage (%)	24 (12.3)	22 (12.3)	0.99
In hospital death (%)	1 (0.7)	1 (0.7)	0.95
Success rate of CMP (%)	140 (72.1)	139 (78.0)	0.18

*CMP* clinical management pathway

**Fig. 1:**
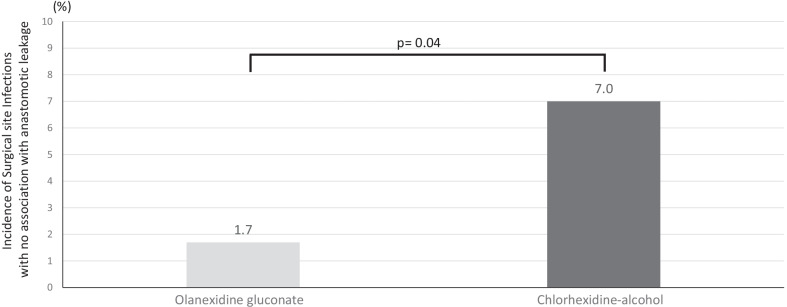
Incidence of surgical site infection (SSI) without anastomotic leakage. Although there were no significant differences between groups in the incidence of SSI without anastomotic leakage, patients prepared with olanexidine gluconate had a lower incidence of SSI

The patients’ variables after propensity score matching are shown in Table 4. There were no significant differences between groups in age, sex, body mass index, ASA grade, preoperative treatment, clinical stage of the disease, and type of surgical approaches. Postoperative events and surgical outcomes are shown in Table 5. There were no significant differences between the matched groups in the total incidence of SSI (12.0% in the chlorhexidine-alcohol group versus 20.1% in the olanexidine gluconate group, *p* = 0.09), incidence of incisional or deep SSI (incisional SSI: 0.8% in the chlorhexidine-alcohol group versus 0.8% in the olanexidine gluconate group, *p* = 0.99; deep fascial SSI: 1.7% in the chlorhexidine-alcohol group versus 3.5% in the olanexidine gluconate group, *p* = 0.39; organ space SSI: 10.3% in the chlorhexidine-alcohol group versus 15.7% in the olanexidine gluconate group, *p* = 0.30; organ space SSI without anastomotic leakage: 0% in the chlorhexidine-alcohol group versus 2.6% in the olanexidine gluconate group, *p* = 0.23). There were no difference in median postoperative hospital stay (p = 0.88). There were no in-hospital deaths in either group, and the success rates of the CMPs were similar in both groups (72.4% in the chlorhexidine-alcohol group versus 74.5% in the olanexidine gluconate group; *p* = 0.71). Notably, there was significant difference between groups in the incidence of all SSI with no association with anastomotic leakage (1.7% in the chlorhexidine-alcohol group versus 7.0% in the olanexidine gluconate group, *p* = 0.04) (Fig. 1).

**Table 4:** Patients’ characteristics in the propensity-matched cohort

Variables	Olanexidine group (n = 116)	Chlorhexidine group (n = 114)	p value
Age (mean ± S.D.)	67.9 ± 3.7	67.8 ± 4.4	0.51
Gender (M:F)	103:13	101:13	0.96
Body Mass Index (mean ± S.D.)	22.1 ± 2.2	21.9 ± 2.3	0.61
ASA Grade			0.47
Grade 1	45	39	
Grade 2	71	75	
Pre-operative treatment			0.13
Chemotherapy	71	47	
Chemo-radiation	8	11	
Clinical stage (UICC 7th)			0.99
Stage I	29	30	
Stage II	20	22	
Stage III	59	55	
Stage IV	8	7	
Type of surgical approach			0.47
Thoracoscopic	98	100	
Thoracotomy	18	14	
Type of surgical approach			0.56
Laparoscopic	101	103	
Laparotomy	15	11

**Table 5:** Postoperative events and success rate of the CMP in the propensity-matched cohort

Variables	Olanexidine group (n = 116)	Chlorhexidine group (n = 114)	p value
30-days SSI (%)	14 (12.0)	23 (20.1)	0.09
Incisional SSI (%)	1 (0.8)	1 (0.8)	0.99
Deep SSI (%)	2 (1.7)	4 (3.5)	0.39
Organ space SSI (%)	12 (10.3)	18 (15.7)	0.30
Organ space SSI without anastomotic leakage (%)	0 (0)	3 (2.6)	0.23
Anastomotic leakage (%)	12 (10.3)	15 (13.1)	0.50
Postoperative hospital stay (median ± IQR)	15.0 ± 3.8	15.0 ± 4.2	0.88
In hospital death (%)	0 (0)	0( 0)	-
Success rate of CMP (%)	84 (72.4)	85 (74.5)	0.71

*CMP* clinical management pathway

## Discussion

After the development of anesthetic techniques, the surgical field was poised to expand the number of operative procedures that could be performed safely. Among the perioperative surgical management factors, optimization of skin antiseptic agents is considered to be of great importance in reducing SSI. Povidone iodine and chlorhexidine have been widely used as skin antiseptic agents in various types of surgery for many years. The efficacy of these agents was investigated extensively in many types of procedures, including surgery and catheter-associated medical procedures [11]. Several clinical studies suggest that chlorhexidine-alcohol reduces the incidence of SSI in comparison with povidone iodine in clean-contaminated surgery [5, 12]. However, although numerous clinical studies have examined which antiseptic skin agent is more effective in preventing SSI, there is still no robust consensus [13].

Olanexidine gluconate is a new antiseptic skin agent developed and approved in Japan. Olanexidine gluconate has antimicrobial activity against a wide range of bacterial species, including Gram-positive and Gram-negative bacteria [7, 14]. Furthermore, olanexidine gluconate reportedly has higher antimicrobial activity against drug-resistant species (such as methicillin-resistant *S. aureus* and vancomycin-resistant *Enterococci*) than other antiseptic skin agents, including chlorhexidine-alcohol or povidone iodine [7, 8]. Several clinical trials have shown the efficacy of olanexidine gluconate in various types of surgery [15–17]. A recent randomized control study reported that the 30-day incidence of SSI in various types of clean-contaminated surgery was 7% in the olanexidine group and 13.3% in the povidone iodine group (adjusted risk reduction: 0.48, 90% CI 0.30–0.74; *p* = 0.002), while there were no significant intergroup differences in the incidence of deep incisional SSI (adjusted risk reduction: 0.55, 90% CI 0.29–1.03; *p* = 0.06) [18]; therefore, the authors concluded that olanexidine gluconate is significantly associated with a low incidence of superficial SSI. However, to our knowledge, no study has investigated the efficacy of olanexidine gluconate in comparison with chlorhexidine-alcohol in clean-contaminated surgery.

It is widely recognized that there are several types of surgical procedures that have accompanying relatively high incidences of SSI in digestive surgery. Patients who undergo thoracic esophagectomy have a higher risk of SSI than those who undergo other types of digestive surgery [19, 20]. In the present study, using a propensity-matched cohort, we compared the incidence of SSI after thoracic esophagectomy with olanexidine gluconate or chlorhexidine-alcohol as the antiseptic skin agent. Although there was no significant intergroup difference in the overall incidence of SSI, the incidence of SSI tended to be lower in patients prepared with olanexidine gluconate (12.0%) than those prepared with chlorhexidine-alcohol (20.1%). It is widely recognized that anastomotic leakage in thoracic esophagectomy is associated with multiple factors, such as patient nutrition and anatomical factors, which are usually unrelated to the skin preparation with antiseptic agents. Therefore, we further investigated the incidence of SSI without anastomotic leakage. Notably, olanexidine gluconate significant reduced the incidence of SSI with no association with anastomotic leakage in comparison with chlorhexidine-alcohol. However, there were no difference in the postoperative hospital stay. Since esophageal cancer surgery is highly invasive, the length of hospital stay for SSI management is not expected to have a significant overall impact in hospital stay unless it is caused by anastomotic leakage. The results were consistent with the overall incidence of SSI in that patients prepared with olanexidine gluconate tended to have a lower incidence of SSI without anastomotic leakage than those prepared with chlorhexidine-alcohol. We could not clearly describe the reason of the superiority of olanexidine gluconate. However, the results of previous clinical studies suggest the superiority of olanexidine gluconate, which is consistent with the results of this study. In addition, previous results of basic research has shown that olanexidine gluconate has high biological activity, especially against drug-resistant bacteria, which may help to interpret the results of this study.

The present study has several limitations. First, this study was investigated retrospectively. A recent randomized control study revealed that olanexidine gluconate significantly reduces the occurrence of overall SSI and superficial incisional SSI compared with povidone iodine in clean-contaminated surgery [18]. Therefore, further study is required to fully reveal the superiority of olanexidine gluconate in comparison with chlorhexidine-alcohol. Second, this study covered a period of more than 2 years, during which time the thoracic and abdominal surgical devices differed slightly. However, the anesthesia and perioperative patient management procedures were performed consistently with the same CMP, which may be considered a strength of the study. Additionally, the prevalence of preoperative treatment was slightly lower in the chlorhexidine-alcohol group than in the olanexidine group (*p* = 0.13). Usually, patients who undergo preoperative treatment have a lower white blood cell count, which potentially makes them more vulnerable to bacterial infections [21]. However, the white blood cell count before surgery did not significantly differ between groups (data not shown). Furthermore, even though more patients were treated preoperatively in the olanexidine gluconate group than the chlorhexidine-alcohol group, the incidences of SSI were not increased.

## Conclusions

In conclusion, present study firstly report the direct comparison between olanexidine gluconate and chlorhexidine-alcohol. The present results indicate that olanexidine gluconate as an antiseptic agent is effective for the prevention of surgical site infectious complications after thoracic esophagectomy with three-field lymph node dissection. Further analysis of a larger number of cases in a randomized controlled study would provide detailed results on the potential influence of olanexidine gluconate in thoracic esophagectomy.

## Data Availability

The datasets used and/or analysis during the current study are available from the corresponding author on reasonable request.

## Abbreviations

SSI: Surgical site infection
POD: Post-operative day
CMP: Clinical management pathway
